# Antiviral Protection by IFITM3 In Vivo

**DOI:** 10.1007/s40588-018-0103-0

**Published:** 2018-08-03

**Authors:** Ashley Zani, Jacob S. Yount

**Affiliations:** 1 Department of Microbial Infection and Immunity, Infectious, Diseases Institute, The Ohio State University, 460 W 12th Ave, Biomedical Research Tower 790, Columbus, OH 43210, USA

**Keywords:** Interferon, ISG, IFITM, rs12252, rs34481144, Virus

## Abstract

**Purpose of Review:**

Interferon-induced transmembrane protein 3 (IFITM3) is a cellular restriction factor that blocks fusion between virus and host membranes. Here, we provide an introduction to IFITM3 and the biochemical regulation underlying its antiviral activity. Further, we analyze and summarize the published literature examining phenotypes of IFITM3 knockout mice upon infections with viral pathogens and discuss the controversial association between single nucleotide polymorphisms (SNPs) in the human *IFITM3* gene and severe virus infections.

**Recent Findings:**

Recent publications show that IFITM3 knockout mice experience more severe pathologies than wild-type mice in diverse virus infections, including infections with influenza A virus, West Nile virus, Chikungunya virus, Venezuelan equine encephalitis virus, respiratory syncytial virus, and cytomegalovirus. Likewise, numerous studies of humans of Chinese ancestry have associated the *IFITM3* SNP rs12252-C with severe influenza virus infections, though examinations of other populations, such as Europeans, in which this SNP is rare, have largely failed to identify an association with severe infections. A second SNP, rs34481144-A, found in the human *IFITM3* promoter has also recently been reported to be a risk allele for severe influenza virus infections.

**Summary:**

There is significant evidence for a protective role of IFITM3 against virus infections in both mice and humans, though additional work is required to identify the range of pathogens restricted by IFITM3 and the mechanisms by which human SNPs affect IFITM3 levels or functionality.

## Introduction

The interferon (IFN)-induced transmembrane proteins (IFITMs) 1, 2, and 3 are upregulated upon stimulation of cells by type I IFNs [1, 2], and these proteins subsequently block membrane fusion between virus and host membranes [3–5]. IFITM cDNAs were among the first IFN stimulated genes (ISGs) to be cloned and sequenced in the early 1980s [6]. However, the ability of IFITM3 to inhibit virus infections was not demonstrated until 2009 when researchers performing a genome-wide siRNA screen for influenza virus host dependency factors conversely discovered that IFITM3 knock-down resulted in increased influenza virus infection of cells [1]. Confirmatory reports were quickly published by several groups in which other screening studies converged upon the importance of IFITMs, and particularly IFITM3, in innate antiviral defense [2, 7–9]. Overexpressed IFITM3 potently inhibits influenza virus infection, and IFITM3 KO cells are highly susceptible to infection [1, 3, 10–13]. Remarkably, IFITM3 KO cells remain highly susceptible to influenza virus infection even after IFN treatments that make WT cells refractory to infection [13]. This in vitro work established that IFITM3 is an essential ISG for cellular defense against influenza virus and, further, that endogenous IFITMs 1 and 2, despite similar antiviral activities to IFITM3 when overexpressed [14], cannot compensate for loss of IFITM3.

IFITM3 is a 15-kDa membrane-associated protein that localizes primarily to endosomes and lysosomes. Evolutionary analysis indicates that an IFITM gene was acquired via horizontal gene transfer from bacteria to a single-celled mammalian ancestor [15]. Though the function of IFITMs in bacteria is entirely unexplored, expression of mycobacterial IFITMs in human cells provided mild resistance to influenza virus infection, suggesting that acquisition of an IFITM gene may have provided an evolutionary advantage against virus infections [16]. IFITM gene duplications are commonly observed in mammalian species[15,17]and have been proposed to provide increased antiviral coverage of cell membranes by maintaining a highly conserved antiviral core amino acid region with divergent N- and C-termini that dictate protein localization and interactions [18]. Indeed, among the long list of viruses that have now been shown to be restricted by IFITM3, a commonality has emerged in that their membrane fusion reactions generally occur in endosomes where IFITM3 is most abundant [19, 20]. IFITM variants that show altered localization may restrict membrane fusion of a distinct subset of viruses, such as those that fuse at the plasma membrane [18, 21, 22].

## IFITM3 Posttranslational Regulation

**Fig. 1:**
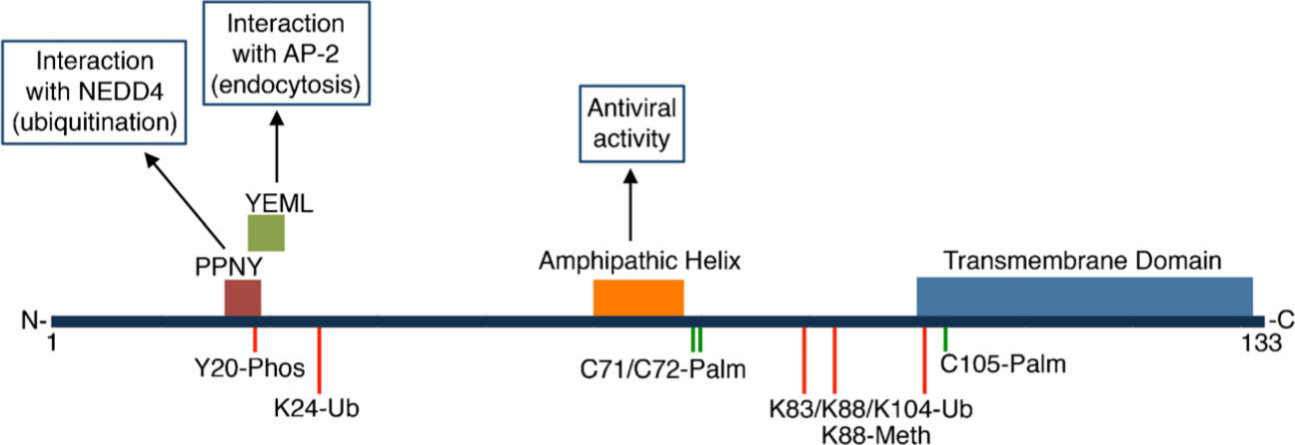
Human IFITM3 critical domains and posttranslational modification sites. The horizontal line represents the 133 amino acids of human IFITM3 from the N- to C-terminus. Colored boxes above the horizontal line represent known structural motifs or domains, and arrows point to characterized functions of these regions. Specific amino acid residues known to be posttranslationally modified are indicated below the horizontal line. Red lines indicate negative regulatory modifications and green lines indicate activating modifications. Phos, phosphorylation; Ub, ubiquitination; Palm, palmitoylation; Meth, monomethylation

The study of posttranslational modifications of IFITM3 has provided numerous insights into its cellular trafficking, turnover/stability, and mechanism of action [23]. IFITM3 undergoes at least four distinct modifications on multiple amino acid residues (Fig. 1). (1) *Phosphorylation* at the IFITM3 N-terminal region on Y20 blocks the interaction of a YxxΦΦ trafficking motif (20-YEML-23 in human IFITM3) with the AP-2 endocytic adaptor protein complex that normally facilitates endocytosis of IFITM3 from the plasma membrane to endosomes and lysosomes (Fig. 1) [21, 24, 25]. Y20 phosphorylation is mediated by the Src family kinase Fyn and results in accumulation of IFITM3 at the plasma membrane and decreased antiviral activity against viruses, such as influenza virus, that enter cells through endocytosis and fuse with endosomes [24, 25]. While phosphorylation may decrease IFITM3 activity against influenza virus, the altered localization enhances its ability to restrict certain viruses that fuse at the plasma membrane as mentioned above [18, 21, 22]. Phosphorylation of IFITM3 is dynamic, involving currently unidentified phosphatases [24, 25]. Though its study led to a better understanding of IFITM3 trafficking, the relevant physiological triggers of phosphorylation and dephosphorylation remain to be identified. (2) *Monomethylation* of IFITM3 at K88 by the SET7 methyltransferase decreases its antiviral activity [26]. This methylation can also be removed by the histone demethylase LSD1, which is recruited to IFITM3 during IFN treatment of cells [27]. Additional studies will be required to determine the molecular mechanism by which K88 methylation inhibits IFITM3 and whether this inhibition is beneficial to cells. (3) *Ubiquitination* of IFITM3 can occur on each of four lysines within IFITM3 (K24, K83, K88, and K104) [11] and is primarily mediated by interaction with the E3 ubiquitin ligase NEDD4 via a conserved PPxY motif in IFITM3 (17-PPNY-20 in human IFITM3) (Fig. 1) [28]. Mutation of the IFITM3 ubiquitination sites or depletion of NEDD4 from cells results in increased stability of IFITM3 [11, 28]. As such, NEDD4 KO cells accumulate high levels of basal IFITM3 even in the absence of IFN stimulation and are thus resistant to infection with IFITM3-sensitive viruses [22, 28]. It is unknown whether IFITM3 is deubiquitinated in cells, and like methylation, it is also unclear whether the negative regulation of IFITM3 via ubiquitination is beneficial to cells given that these modifications limit the antiviral function of IFITM3. (4) *S-palmitoylation* of IFITM3 occurs on three highly conserved cysteine residues (C71, C72, and C105) and is the only modification discovered to date that is required for robust IFITM3 antiviral activity [2, 11, 29–31]. This lipid modification is constitutively added to IFITM3 in an irreversible manner [11] by at least three cellular palmitoyltransferase enzymes (ZDHHC3, ZDHHC7, and ZDHHC20) [13]. Nearly 100% of IFITM3 is palmitoylated on at least one cysteine, and examination of antiviral activity of IFITM3 mutants lacking specific cysteines suggests that C72 is particularly critical for inhibition of influenza virus infection [32]. The primary function of *S*-palmitoylation at C72 and the neighboring C71 is likely the targeting or anchoring of a highly conserved amphipathic helix (59-VWSLFNTLFM-68 in human IFITM3) that is required for inhibition of membrane fusion by IFITM3 and that is adjacent to these *S*-palmitoylation sites in the IFITM3 amino acid sequence (Fig. 1) [33•].

## IFITM3 Mechanisms of Action

The primary mechanism by which IFITM3 inhibits virus infection is via blockade of fusion pore formation between virus and host membranes [3, 5]. This prevents the entry of viral genomes into the cytosol and subsequent virus replication. Single particle imaging of fluorescently labeled viruses has revealed that hemifusion between virus and host membranes occurs in cells expressing IFITM3, but that the fusion process is stalled prior to formation of a membrane pore [4]. Insertion of the aforementioned IFITM3 *S*-palmitoylated amphipathic helix into the cytoplasm-facing leaflet of the host membrane bilayer has been proposed to stabilize a membrane intermediate of the fusion process, consistent with the well-characterized ability of amphipathic helices to induce or stabilize membrane curvature [33•].

The inhibition of fusion of incoming virus requires that IFITM3 is induced and present at inhibitory levels in the target cells. However, IFITMs may have additional functions subsequent to initial infection [34]. Groups studying HIV infections first showed that IFITMs induced in infected cells are incorporated into the membrane envelopes of nascent virions and that this presence of IFITMs limits virion infectivity [35, 36]. This effect was subsequently shown for numerous additional viruses and appears to be a wide-ranging additional function of IFITMs, including IFITM3 [37]. IFITMs in virions likely impair fusogenicity of the viruses, and this likely also involves the conserved amphipathic helix. Additional experiments are required to determine precisely how IFITM3 affects membrane fusion dynamics, and such experiments will not only be informative as to the mechanisms of action of IFITM3, but will also shed light on the fundamental fusion mechanisms used by enveloped viruses.

## IFITM3 Limits Severity of Virus Infections in Mice

IFITM3 KO mice were initially generated to study a suspected role of IFITM3 in germ cell development in vivo [38]. However, neither IFITM3 KO mice nor mice lacking the entire IFITM locus (referred to as IFITMdel mice in the literature) possessed a defect in germ cells or reproduction [38]. Upon discovery of the antiviral activity of IFITM3 in vitro, these mice were re-examined for phenotypes during microbial infections (Table 1). IFITM3 KO mice were observed in two studies to experience increased weight loss and mortality from H1N1 and H3N2 influenza A virus infections, correlating with increased virus lung titers, systemic lymphopenia, and increased pro-inflammatory cytokine levels in the lungs [39, 40]. Remarkably, IFITMdel mice did not experience more severe infections than IFITM3 KO mice, suggesting that other IFITMs do not contribute significantly to influenza virus resistance in vivo [40]. In addition to the role of IFITM3 in the innate immune response, it also protects lung dendritic cells from productive infection with influenza virus, thus allowing their trafficking to draining lymph nodes and priming of CD8 T cells [46]. IFITM3 also protects resident memory CD8 T cells from infection and death in the lung during secondary influenza virus infections [47]. Thus, the importance of IFITM3 in primary and secondary influenza virus infections is multifactorial.

Additional viruses that show susceptibility to IFITM3 inhibition in vitro have also been examined in IFITM3 KO mice. Chikungunya virus infection in the footpad caused increased ankle joint swelling in IFITM3 KO versus WT mice, correlating with increased virus burden in the serum, spleen, and ankle early in infection [41•]. Similarly, IFITM3 KO mice experienced increased lethality upon footpad infection with Venezuelan equine encephalitis virus that was accompanied by increased virus titers in the spleen, liver, spinal cord, and, most significantly, brain [41•]. Together, these experiments demonstrate that IFITM3 is essential for limiting the severity of alphavirus infections in vivo.

Several flaviviruses, such as Dengue virus, West Nile virus, Zika virus, and hepatitis C virus, are also inhibited by IFITM3 in cell culture models [1, 48–51]. In vivo, subcutaneous infection with West Nile virus induced increased lethality in IFITM3 KO mice compared to that in WT mice [42]. This enhanced pathogenicity correlated with increased virus titers in the serum, lymph node, brain, and spinal cord. Using bone marrow reconstitutions, it was determined that IFITM3 plays essential roles in both hematopoietic and non-hematopoietic cells to restrict West Nile virus infection severity. Interestingly, unlike subcutaneous infection, intracranial infection did not result in significant differences in virus burden in the brain or spinal cord, suggesting that IFITM3 does not directly inhibit West Nile virus in the central nervous system [42].

IFITM3 KO mice also showed modest weight loss during respiratory syncytial virus infection that was absent in WT mice [45], consistent with effects of IFITM3 on this virus in vitro [44]. This weight loss correlated with increased levels of virus in the lungs and increased lymphocyte counts in lung tissue and lung lavage fluid of KO mice [45]. In general, mice are considered a poor model for respiratory syncytial virus infection since high doses of virus are required to achieve measurable infection of lung cells and since weight loss indicative of illness is not usually observed. The small amount of weight loss observed in IFITM3 KO mice suggests that IFITM3 may contribute to respiratory syncytial virus susceptibility in vivo [45], but its loss does not overcome the significant species restriction barrier for this virus in mice.

IFITM3 may also provide a protective effect in certain infections independent of its inhibition of virus entry into cells. Examination of cytomegalovirus infection of several cell types in vitro indicated that this virus is not restricted by IFITM3 [43•]. However, IFITM3 KO mice experience more severe weight loss and increased mortality compared to WT mice following intraperitoneal cytomegalovirus infection [43•]. These effects correlated with a general lymphopenia in circulation and in the spleens of KO mice early in infection. Virus burden and IL-6, TNF, and IFNα were concurrently increased in these spleen homogenates. Stimulation of IFITM3 KO dendritic cells in vitro with irradiated cytomegalovirus or Toll-like receptor ligands resulted in increased secretion of IL-6 compared to that of WT cells. Further, treatment of infected WT or KO mice with anti-IL-6 neutralizing antibodies caused a reduction in weight loss during infection and also restored NK cells to WT levels in the KO mice [43•]. Interestingly, IL-6 was also among the cytokines increased in the lungs of influenza virus-infected IFITM3 KO mice [39]. Overall, these results suggest that IFITM3 may possess a mechanistically uncharacterized ability to dampen IL-6 production [43•] and also that IFITM3 may play underappreciated roles in preventing cytokine-driven immunopathologies, even in infections that are not directly restricted by IFITM3.

## *IFITM3* Polymorphisms in Humans Are Linked to Severe Virus Infections

The well-defined ability of IFITM3 to prevent infection of cells by influenza virus, along with the increased virus-induced morbidity and mortality of IFITM3 KO mice, has prompted numerous research groups to examine whether single nucleotide polymorphisms (SNPs) in the human *IFITM3* gene associate with influenza virus infections or infection severity (all studies to date are summarized in Table 2). The pioneering study in this field sequenced the *IFITM3* gene regions of 53 patients hospitalized with severe 2009 H1N1 pandemic influenza A virus infection [39]. Three of these patients were homozygous for a SNP, rs12252-C, where the T majority allele is substituted with a C. Three out of 53 was a significant overrepresentation of the C allele as compared to matched European controls from 1000 genomes in which the SNP is rare. The authors of this study hypothesized that the C substitution alters a splice acceptor site, resulting in a truncated IFITM3 protein lacking its first 21 amino acids. Overexpression of the truncated IFITM3 variant provided less inhibition of influenza virus as compared to WT IFITM3 [39], and subsequent studies demonstrated that this mutant is mislocalized due to loss of its YxxΦ endocytosis motif as discussed above [18, 21, 24, 25]. While altered splicing and mislocalization of IFITM3 provided a satisfying mechanistic explanation for effects of the rs12252-C SNP, the existence of truncated IFITM3 protein has not been observed in cells homozygous for the C allele [39, 66•]. Likewise, studies in which RNA sequencing was performed reported that the predicted alternatively spliced transcripts were not detected in cells from individuals homozygous for the C allele [58, 66•]. Thus, whether there is a direct effect of rs12252-C on IFITM3 function or influenza virus infections remains unclear.

Several follow-up studies examining Europeans and other populations in which rs12252-C is rare have failed to find an association between the SNP and severe influenza virus infection [57–62], although some studies support a link between the SNP and mild influenza [56, 57]. In contrast, a strong overrepresentation of homozygous rs12252-C genotypes in severe influenza patients has been repeatedly observed in studies examining Chinese individuals in which the allele frequency for this SNP is greater than 50% in the general population [52–55]. This association is consistent between studies examining 2009 H1N1 pandemic virus [53, 55], H7N9 virus [53, 54], and seasonal influenza virus [52] infections. Likewise, Chinese individuals who were infected with Hantaan virus and experienced severe hemorrhagic fever were also more likely to be carriers or homozygous for rs12252-C than the general Chinese population [63]. A study examining HIV-infected Chinese individuals also reported that carriage of rs12252-C was more common among rapid progressors with higher viral load set points and a faster decline of CD4 T cells than in those with slower progression of disease [64]. However, results from this study have been cautiously interpreted by the field and will require additional corroboration since rs12252-C homozygous individuals were surprisingly underrepresented among the rapid progressors [64]. In sum, there is generally a strong association between severe influenza virus infections and homozygosity of the *IFITM3* rs12252-C SNP in Chinese individuals, while infrequency of the SNP in Europeans makes it difficult to confidently assess its effects in this population (Table 2).

A recent landmark study examined a second *IFITM3* SNP located within the IFITM3 gene promoter [65]. A minor allele, rs34481144-A, was associated with severe influenza virus infections in a largely African American population and with death following influenza virus infection in a largely European population [65••]. Additionally, early replication of influenza virus in a third study group made up of individuals of European ancestry was also linked to carriage of this SNP [65••]. In the populations examined, this SNP ranged in allele frequency from 16.9 to 33.25%. Mechanistically, cells from individuals possessing the rs34481144-A SNP showed decreased *IFITM3* mRNA and protein levels in a dose-responsive manner, correlating with the presence of one or two rs34481144-A alleles [65••]. This effect on *IFITM3* gene expression was suggested to be due to differential occupancy of the *IFITM3* promoter by activating versus inhibitory transcription factors [65••]. These intriguing new findings further support the importance of IFITM3 in antiviral defense in vivo, and indeed, SNPs in *IFITM3* remain the only reproducibly observed human genetic associations with severe influenza virus infections [67].

## Remaining Questions

We have only begun to understand the importance and functions of IFITM3 in antiviral defense in vivo. For example, only a limited number of the viruses restricted by IFITM3 in vitro have been studied in IFITM3 KO mice and even fewer infections have been examined in humans. To date, studies in humans have primarily focused on influenza virus infections. The discovery of the importance of the rs34481144-A SNP in controlling IFITM3 levels [65••] should facilitate the study of IFITM3 in additional human infections in diverse populations. Additionally, the discovery of a regulatory role for IFITM3 in IL-6 production [43•], along with a recent report that IFITM3 is a negative feedback inhibitor of the type I IFN induction pathway [68], may suggest that IFITM3 is a broad regulator of inflammation and that it may play important roles in infections in which the balance between pathogen control versus tissue pathology is determined by these cytokines. Interestingly, IFITM3 KO mice were not observed to be more susceptible to infections with *Salmonella typhimurium*, *Citrobacter rodentium, Mycobacterium tuberculosis, or Plasmodium berghei* [45] (Table 1). Thus, at present, the critical role of IFITM3 in antimicrobial defense appears to be limited to virus infections, though this remains an open question.

Given the strong and reproducible association between homozygosity of the *IFITM3* rs12252-C SNP and severe influenza in the Chinese population, it will be important to determine the underlying mechanism by which this SNP influences disease. The initially proposed hypothesis that the SNP affects splicing [39] has not been supported by subsequent studies [58, 66•]. Whether rs12252-C influences *IFITM3* gene splicing or protein levels in a cell type-specific manner and whether this SNP cosegregates with a different causative allele both remain unknown.

The antiviral effects of IFITM3 are reversed by the membrane-destabilizing drug amphotericin B, which is commonly prescribed to treat fungal infections in humans [69]. Mice treated with amphotericin B experience more severe influenza virus infections, similar to IFITM3 KO mice [69]. We recently determined that IFITM3 is the first known restriction factor for human metapneumovirus infection, and we confirmed that amphotericin B completely neutralizes the antiviral activity of IFITM3 against both influenza virus and human metapneumovirus [22]. Thus, it will be interesting and important to determine whether clinical use of amphotericin B has the unintended consequence of increasing susceptibility to or pathogenicity of these and other virus infections due to its neutralizing effects on IFITM3.

Antiviral restriction factors often drive the evolution of viruses to evade or inhibit virus restriction [70]. IFITM3 inhibits the membrane fusion of influenza virus, which is typically triggered by low pH in endosomes [3–5]. Given that endocytosis and membrane fusion are fundamental steps in the entry of all influenza viruses, all strains of the virus that have been tested to date are susceptible to IFITM3 restriction to varying degrees. The degree of susceptibility appears to be determined by the pH optimum at which the virus fuses. Viruses that fuse at low pH in late endosomes where IFITM3 is abundant are strongly restricted [71, 72]. Conversely, viruses that have evolved to fuse at a higher pH in early endosomes where IFITM3 is less abundant are partially resistant to IFITM3 restriction [71, 72]. Similarly, in vitro studies of HIV have shown that over time in culture the HIV env protein will evolve to evade restriction of the virus by IFITMs [73]. Lack of restriction by IFITMs is indeed a characteristic of transmitted founder HIV isolates derived from recently infected humans [74••, 75]. Interestingly, as HIV evolves its env protein in vivo to evade adaptive immune responses, restriction by IFITMs increases [74••, 75]. The on-going evolutionary battle between viruses and IFITMs will be an exciting area for further investigation.

## Conclusions

Several broad conclusions can be drawn from the research performed on IFITM3 over the past decade. First, IFITM3 is a broad inhibitor of membrane fusion between viruses and cell membranes and is particularly active against viruses that enter cells via endocytosis due to its primary localization at endosomes. Second, IFITM3 utilizes an amphipathic helix to block virus membrane fusion. Third, IFITM3 is regulated by multiple posttranslational modifications that determine its intracellular localization, activity, and abundance. Fourth, IFITM3 knockout mice experience increased susceptibility to a wide range of virus infections. Fifth, *IFITM3* gene SNPs in the human population are correlated with severe virus infections. The continued study of IFITM3 and other IFITMs in terms of their precise mechanisms of action, their range of microbial restriction in vivo, effects of human SNPs, and possible viral countermeasures will continue to increase our understanding of virus-host interactions.

## Figures and Tables

**Table 1:** Summary of pathogens examined in IFITM3 KO mice and whether or not increased pathogenicity was observed compared to WT mice

Pathogen	Increased pathogenicity in IFITM3 KO mice	Reference
Influenza A virus H1N1	Yes	[39, 40]
Influenza A virus H3N2	Yes	[39]
Chikungunya virus	Yes	[41•]
Venezuelan equine encephalitis virus	Yes	[41•]
West Nile Virus	Yes	[42]
Cytomegalovirus	Yes	[43•]
Respiratory syncytial virus	Yes	[44]
*Salmonella* typhimurium	No	[45]
*Citrobacter rodentium*	No	[45]
*Mycobacterium tuberculosis*	No	[45]
*Plasmodium berghei*	No	[45]

**Table 2:** Summary of human IFITM3 SNP studies. Assoc w/, association with; *Pop.,* population; *Al,* allele; *Homo.,* homozygous; *Chin,* Chinese; *Iran,* Iranian; *Eur,* European; *Afr,* African American; *Span,* Spanish; *Port,* Portuguese; *Flu (S),* seasonal influenza virus;*Flu (H7),* H7N9 influenza virus; *Flu (HI),* 2009 H1N1 pandemic influenza virus; *Flu (H3),* H3N2 influenza virus; *Hosp,* hospitalization; *Prog,* progressor; *Rep,* replication; *1000 G,* 1000 genomes; *Flu-Neg,* influenza virus-negative

SNP	Assoc w/disease	Study Pop.	Virus	Disease	Control Pop.	SNP A1.% controls	SNP A1.% cases	SNP Homo. % controls	SNP Homo. % cases	# cases	Ref
rsl2252-C	Yes	Chin	Flu (S)	Severe flu	Healthy	49.2	76.5	23	61.5	164	[52]
	Yes	Chin	Flu (H7)	Death	Survivors	51.6	65	22.6	50	10	[53]
	Yes	Chin	Flu (HI)	Death	Survivors	55.8	69.5	35	56.5	23	[53]
	Yes	Chin	Flu (H7)	Hosp	1000 G	52	59.4	26	37.5	16	[54]
	Yes	Chin	Flu (H1)	Severe flu	1000 G	50.2	81.2	25.3	68.7	52	[55]
	Yes	Iran	Flu (S)	Mild flu	Flu-Neg	2.4	9.49	0.8	3.8	79	[56]
	Yes	Eur	Flu (H1)	Severe flu	1000 G	3.4	9.4	0.3	5.7	53	[39]
	Yes	Eur	Flu (S)	Mild flu	Healthy	4	5.02	0.15	0.7	259	[57]
	No	Eur	Flu (S)	Severe flu	Healthy controls	4	4.4	0.15	0	34	[57]
	No	Eur	Flu (S)	Severe flu	1000 G	4.1	3.8	0	1.1	185	[58]
	No	Afr	Flu (S)	Severe flu	1000 G	26.1	25.8	7	7.1	56	[58]
	No	Eur	Flu (S)	Hosp	Healthy	2.9–3.5	2.9–1.2	0–0.1	0–0.9	238	[59]
	No	Span	Flu (H1)	Hosp	Healthy	3.5	5.8	0	0	60	[60]
	No	Port	Flu (H1)	Hosp	Infected non-Hosp	8.7	7.7	0	2.4	84	[61]
	No	Port	Flu (H1)	Severe flu	Healthy	6	9	0	0	22	[62]
	Yes	Chin	Hantaan	Severe Hem Fever	1000 G	52.1	68.2	26.9	43.9	41	[63]
	Yes	Chin	HIV-1	Rapid Prog	Normal Prog	52.7	60.8	35.2	29.7	74	[64]
rs34481144-A	Yes	19% Eur, 81% Afr	Flu (H1)	Severe flu	Infected mild cases	16.9	49.9	1.3	33.3	9	[65••]
	Yes	Eur	Flu (H3)	Early virus Rep	Infected controls	28.2	55.3	4.3	21.1	19	[65••]
	Yes	73% Eur, 17% Afr	Flu (S)	Death	Infected survivor	33.2	49.9	14.1	17.6	18	[65••]
